# Artificial vibrotactile feedback elicits neural correlates of sense of agency

**DOI:** 10.1186/s12984-025-01850-2

**Published:** 2026-01-07

**Authors:** Inés Martín Muñoz, Nicolas Berberich, Gordon Cheng, Agnieszka Wykowska

**Affiliations:** 1 Institute for Advanced Study, Technical University of Munich, Arcisstraße 21, 80333 Munich, Germany; 2 Institute for Cognitive Systems, Technical University of Munich, Arcisstraße 21, 80333 Munich, Germany; 3 Social Cognition in Human-Robot Interaction, Italian Institute of Technology, Via Morego 30, Genova, 16163 Italy

**Keywords:** Artificial sensory feedback, Sense of agency (SoA), Agency measurement

## Abstract

**Background:**

The Sense of Agency (SoA) refers to the subjective experience of having control over our own actions and their outcomes. SoA is experienced when there is a match between the predicted and actual sensory outcomes of an intended motor action. Thus, receiving sensory feedback related to motor actions is essential for the formation of the SoA. As a consequence, individuals with sensory loss due to neuropathies or limb impairments might experience reduced SoA. Therefore, incorporating artificial sensory feedback in assistive technologies (e.g. prosthetics, exoskeletons, sensory substitution systems) is crucial for patients to properly experience SoA and thereby enhance the effective use of such technologies. In this study, with the use of neuro-cognitive measures, we validated the effect of a vibrotactile sensory feedback system on SoA for lower-limb movements.

**Methods:**

Healthy participants (*n* = 25) performed a stepping task while receiving vibrotactile sensory feedback and, in a control condition, auditory feedback. We employed SoA measures to validate the sensory feedback system, in particular the N100 ERP component of the electroencephalography (EEG) signal as an implicit neural measure of SoA, temporal interval estimation tasks as an implicit behavioural measure, and SoA questionnaires as an explicit measure. Statistical analyses, including repeated-measures ANOVA and t-tests, were conducted to examine the effects of externally generated versus self-generated stimuli on N100 modulation, temporal interval estimations, and SoA questionnaire results.

**Results:**

Our results showed a significant modulation of the N100 component for self-generated, relative to externally generated, sensory feedback. The direction of the effect was opposite for vibrotactile and auditory sensory feedback, with enhancement in vibrotactile and attenuation in auditory modality. In contrast, no notable effects were found in interval estimation tasks or explicit agency measures, pointing to a possible dissociation between implicit and explicit measures of SoA and indicating higher sensitivity of implicit neural measures.

**Conclusions:**

Sensory processing of the vibrotactile sensory feedback is sensitive to the difference between outcomes that are actively generated compared to passively received, as indexed by the modulation in amplitude of the N100 component. This is an indication that vibrotactile sensory feedback is capable of eliciting sense of agency. Additionally, we found that modulation of sensory processing is more sensitive than temporal interval estimates to self- vs. externally-generated outcomes. Therefore, we suggest using it as a metric for the design and validation of assistive technologies.

## Background

 An emerging topic of interest in the fields of prosthetics, robotic teleoperation, and assistive technology is the sense of agency, the feeling of control over our own actions and their outcomes [1, 2].

Sense of Agency (SoA) relies on the ability to distinguish between self-generated and externally generated events, and it has implications for voluntary motor control, learning, and interactions with the environment. The importance of SoA becomes paramount in clinical cases of people suffering from sensory impairment, and in technical systems that use sensory feedback as a mechanism to enhance user control and performance.

### Sense of agency and the comparator model

A predominantly accepted theoretical model of Sense of Agency is the *comparator model* introduced by Frith et al. [3]. The model explains how the SoA over motor actions and their sensory outcomes is formed by the comparison between the predicted and actual outcome. This comparison results in a match or a mismatch. If a match occurs, the action is processed as successfully self-generated and completed, and SoA is elicited. However, if the actual motor action is disrupted and external forces cause the sensory outcome not to match the planned action outcome, there will be a mismatch between the actual and the predicted sensory outcomes, and no SoA is experienced in relation to the action outcome [4].

A neural mechanism related to this process is *sensory attenuation*. When an action is classified by the brain as self-generated, the sensory processing of the associated action outcome is attenuated, meaning that self-generated stimuli are perceived as less intense than externally generated stimuli [5]. This is presumably due to that self-generated sensory outcomes are better predicted by the brain, relative to externally-generated stimuli, and thus, evoke a weaker response [6, 7]. Sensory attenuation has been investigated using various methods, including both behavioural and neuroimaging studies. The force-matching paradigm quantifies perceptual sensory attenuation by asking participants to match a force applied to their index finger [8]. Results from previous research show that the matched force is overestimated when the participants self-generate the force in comparison to when the force is applied using an external control device [9], which illustrates the sensory attenuation of self-generated sensory outcomes. Another approach show that participants rate the volume of self-generated sounds as lower than externally generated stimuli, which demonstrates sensory attenuation [10, 11]). These behavioural methods rely on the subjective reporting of the participants. However, sensory attenuation effects have also been investigated using neural measures such as functional magnetic resonance imaging (fMRI) [12, 13]. Additionally, electroencephalography (EEG) studies have revealed that cortical activity is modulated depending on whether a stimulus is self-generated, and thus expected, or externally generated and unexpected. Event-related potential (ERP) reflect the electrical activity of pyramidal neurons activated during the processing of sensory stimuli [14]. Previous research has shown that early ERP components, such as N100 at 100 ms, are modulated in relation to the sensorimotor predictability of the stimulus [15–17], while later components, P200 and P300, are typically associated with the outcome evaluation and affective processing and potentially related to SoA as indicated by positive correlations between P300 amplitude and agency measures [18, 19].

Generally, an attenuation of the N100 component is observed in response to self-generated stimuli in comparison to externally generated stimuli [20–22]. This phenomenon is interpreted as neural evidence of sensory prediction and attenuation mechanisms associated with the comparator model and SoA. Most of the research in this area has been conducted using an auditory tone as an action outcome, with predictable outcomes being associated with smaller N100 amplitudes [20]. However, in some instances, sensory enhancement has been observed in situations in which there were increased attentional demands due to contextual factors, such as delayed or low-intensity stimuli [23, 24]. These results show that the processing of sensory stimuli is modulated by whether the stimuli are attributed to one’s own action or to an external source. However, the directionality of this effect (attenuation or enhancement) is not clear and likely depends on additional factors, such as attentional and predictability conditions. Therefore, we will hereafter refer to this effect as “*modulation*” of sensory processing related to SoA.

### Sense of agency measures

Despite the well-established importance of SoA [1, 25–27], quantifying it reliably remains a challenge. SoA can be assessed through both implicit and explicit measures. Explicit SoA is generally evaluated using self-reported questionnaires or verbal reports from individuals over their experienced level of control over an action and its consequences. However, explicit SoA metrics are susceptible to other factors, such as the level of alertness of the individual, the cognitive and physical demands of the experimental task, or other biases [28–30]. On the other hand, implicit SoA measures, such as modulation of sensory processing and temporal interval estimations, provide more quantitative and objective metrics.

Modulation of sensory processing refers to the modulation of the perceptual and neural responses that occur when the predicted and actual outcome of a voluntary action match. Temporal interval estimations, on the other hand, are used as a metric of intentional binding, the phenomenon in which perception of temporal interval between voluntary actions and their sensory outcomes is perceived as compressed [31], relative to involuntary actions or passive control. Shorter temporal intervals are associated with a stronger SoA. However, the reliability of temporal interval estimation as a metric for SoA remains unclear. Some studies have reported shifts in action-effect timing perception under agency conditions, while others have not found significant effects [32]. The inconsistent findings raise the possibility that temporal interval estimation may not be a reliable metric for SoA.

### Sensory substitution and assistive technology

When sensory feedback is disrupted or absent, such as in the case of individuals with polyneuropathy or amputation, the comparator model is compromised, and SoA is weakened. There are compensatory mechanisms, e.g. visual feedback, that can partially mitigate the consequences of sensory loss. But the absence of confirming sensory feedback can severely affect SoA, ultimately impacting the quality of life of these individuals. Thus, assistive technologies (e.g. sensory substitution systems, prostheses, and other wearable technologies) should focus not only on motor functions but also on enhancing SoA. It is crucial for users to effectively integrate the assistive devices and perceive them as extensions (or parts) of one’s body, ensuring that the device is perceived as responsive to one’s intentions [2, 33]. Eliciting and preserving SoA is essential for better functional performance, user adaptation, and more natural and effective use [34, 35].

A technological approach to restore SoA in individuals with sensory impairments is to provide sensory substitution. Sensory substitution replaces a missing or impaired sensory modality with artificial input from another modality or artificial input relocated to a non-affected area. This enables individuals to experience their environment and the sensory outcomes of their own actions through alternative means. Some examples of sensory substitution include the use of auditory or tactile modalities to convey visual information [36, 37]. In the use of prostheses, sensory feedback impacts both the prosthesis’s performance and the user experience when interacting with the prosthesis, promoting a feeling of agency and ownership, and electrical and vibrotactile feedback have been used to help improve the feeling of ownership and to establish SoA [15, 38, 39].

Sensory feedback has been identified as an important factor to consider in the design of prostheses and other assistive devices [40]. Moreover, various sensory modalities are often used, including auditory, visual, electrical, and vibrotactile modalities. Out of these modalities, vibrotactile feedback stands out as a non-invasive and discreet sensory modality, ideal for users that would prefer a non-visible and unobtrusive system [41]. In addition, other user-centred studies suggest that tactile feedback is preferred by users in noisy environments where auditory feedback might be missed [42]. Lastly, vibrotactile stimuli are more proximal to the natural haptic feedback we receive while performing motor actions or manipulating objects, while other feedback such as auditory feedback is more distal. Validating assistive systems, particularly those employing vibrotactile feedback, regarding their impact on SoA would provide valuable information about the user’s perceived sense of control and potential for integration of the system.

In summary, amputees and other individuals with sensory loss can benefit from the use of assistive technologies that incorporate artificial sensory feedback. In addition, and due to the fact that SoA emerges from the match between predicted and actual sensory outcomes, this artificial sensory feedback likely has not only functional benefits but also positive implications in eliciting SoA, where SoA, in turn, should result in higher acceptance rates and increase of general well-being. Furthermore, it would be advantageous to establish a SoA metric that can reliably assess SoA during the use of assistive technologies.

### Aim of study

Vibrotactile and haptic feedback are commonly used in the field of prosthetics and assistive technologies, but this type of feedback is understudied in relation to SoA [43–45]. To address this research gap, in this study, we validated a vibrotactile sensory feedback system in terms of its ability to elicit SoA in users. We designed an experimental paradigm in which healthy participants received either externally generated or self-generated vibrotactile sensory feedback. We asked the participants to perform a stepping action and detected their movement using pressure insoles. The validation of our system was performed against a system that employed standard auditory feedback as in most SoA studies [20, 21, 46, 47]. To assess SoA, we analysed modulation of sensory processing manifested by modulation of the EEG signal, temporal interval estimations, and self-reported ratings.

## Methods

### Experimental design

Our experiment followed a 2 × 3 factorial design in which participants completed two sensory modality blocks with three experimental conditions per block (see Table 1). Each participant completed all conditions in a within-subjects design. The order of sensory modality blocks and of conditions within each block was counterbalanced across participants to control for order effects.

**Table 1:** Overview of the 2 × 3 factorial experimental design.

Auditory modality condition (A)		
Auditory operant condition (OT)	Auditory temporal interval estimation baseline (IEB)	Auditory passive condition (P)
Vibrotactile modality condition (V)		
Vibrotactile operant condition (OT)	Vibrotactile temporal interval estimation baseline (IEB)	Vibrotactile passive condition (P)

The study included two sensory feedback modalities (Auditory and Vibrotactile), each consisting of three conditions: operant task (OT), Temporal interval Estimation baseline (IEB), and passive condition (P). Each participant completed all six conditions. The order of sensory modalities and conditions was counterbalanced across participants to control for order effects

The auditory block (A) and the vibrotactile block (V) were the two sensory modality blocks. We separated the conditions into two blocks to minimize any potential cross-modal interference. Each modality block consisted of three different experimental conditions: the operant task (OT) condition, the temporal interval estimation baseline (passive) (IEB) condition, and the passive (P) condition. In the OT condition, participants performed the voluntary movement at their own pace and subsequently received the sensory stimulus. The stimulus was time-locked to the action with a delay of 200ms, 500ms, or 800ms [48–51]. After the presentation of the stimulus, participants were asked to estimate the temporal interval of time between their action and the sensory stimulus. In the passive IEB condition, participants received the sensory stimulus after a colour change in the visual cue. The period of time between the start of the trial and the change in the visual cue was modelled after the movement onset (mean ± standard deviation) for the voluntary action in the OT condition. The sensory stimulus was time-locked to the change in the visual cue with a delay of 200ms, 500ms, or 800ms. Then, participants were asked to estimate the time interval between the change in the visual cue and the sensory stimulus. This was done as a baseline condition for the temporal estimates that were performed during the experimental (OT) conditions. Lastly, in the P condition, participants passively received the sensory stimulus after a random delay without performing any voluntary action or temporal interval estimation. This condition was introduced as a baseline for the ERP effects in the OT condition.

The P and the OT conditions each had 60 trials per sensory modality each and were used for the EEG data analysis. The IEB condition had 30 trials and was used, together with 30 randomly selected trials from the OT condition, for the temporal interval estimations analysis. This experimental design allowed us to separately control for externally and self-generated stimuli as well as to control for artefacts in the EEG signal due to changes in the visual cue.

An additional motor-only condition was performed during the experiment. In this motor-only condition, participants performed the same step movement as in the operant task condition but without receiving any stimulus in response. The initial purpose of this condition was to correct the EEG data from the operant task condition to remove the step related movement artefacts. However, in the final analysis the muscle artifacts were removed using ICA and the data from this motor-only condition were not needed.

### Participants

Twenty-five healthy adult participants (16 males, mean age = 27.72 years, standard deviation = 7.01 years, range = 18 to 55 years) were recruited for this study. This sample size was selected based on previous studies [23, 52] on SoA and sensory attenuation. All participants were right-handed, had normal or corrected-to-normal vision, and reported no history of neurological or psychiatric disorders.

The study was conducted following the Declaration of Helsinki and was approved by the local ethics committee (TUM Ethics Committee, 2024-10-NM-KH). All participants gave written consent before participating in the experiment and received 10 euros per hour as monetary compensation. If participants inquired, they were debriefed about the experiment’s purpose after completing the task.

### Apparatus and sensory stimulus

The experimental task was presented on a 16-inch laptop screen, with a refresh rate of 144 Hz. The participant stood 50 cm away from the screen. All experimental conditions were implemented in Python (Python 3.11.1) using the Pygame library (version 2.4.0). The participants were shown a neutral grey background in which a white fixation cross was shown.

For the temporal interval estimations, participants were shown a continuous scale from 0 to 1000ms, with guiding marks every 100ms. Participants then used their left hand on the laptop touchpad to click on the corresponding part of the scale, and their response was saved as an integer in a CSV file, together with the actual stimulation delay for that trial.

SoA subjective ratings were collected through a SoA questionnaire at the end of each sensory modality block. For each modality, participants were shown four questions, two regarding their level of control over the stimulus and two regarding their perception of the stimulus (see Supplementary material). Each question was rated with a 7-point Likert scale, from strongly disagree to strongly agree. The questionnaire, as well as the participant data (sex and age) was collected using a locally run webpage, implemented using the Flask library (3.0.3.) in Python.

The auditory stimulus was a pure tone with a duration of 100ms and a frequency of 1000 Hz, generated using Python. The stimulus was sampled at 44.1 kHz and delivered to the right ear using Philips SHP2500 headphones. The volume was set to a comfortable yet sufficiently loud level to ensure clear tone perception. This level was individually calibrated for each participant before the experiment.

The vibrotactile stimulus was a 100ms vibration generated using a coin vibromotor (Polulu Shaftless Vibration Motor). The vibromotor measures 10 mm in diameter and has a vibration amplitude of 0.75 g at 3 V, as reported in the manufacturer’s calibration data sheet. The vibration frequency was approximately 200 Hz. The vibromotor was controlled using a Seeed Studio XIAO nRF52840 micro-controller connected to the experimental computer via UART communication. During the vibrotactile block of the experiment, the vibromotor was placed on the right arm of the participant, over the extensor digitorum muscle, approximately 5 cm above the wrist. Additionally, the stepping area was covered with a soft material and the participants wore noise-cancelling headphones to reduce the sound associated with the footstep, as well as the sound from the vibromotor being activated.

Lastly, we used OpenGo pressure insoles (Moticon, Germany) to detect the stepping movement of the participants. Each insole has 16 pressure sensors and there were five pairs of pressure insoles available to account for differences in shoe sizes. The centre of pressure values were used to detect when the participants performed the required action in the operant-task conditions. The insoles were wirelessly connected to the experimental computer via Bluetooth Low Energy.

**Fig. 1:**
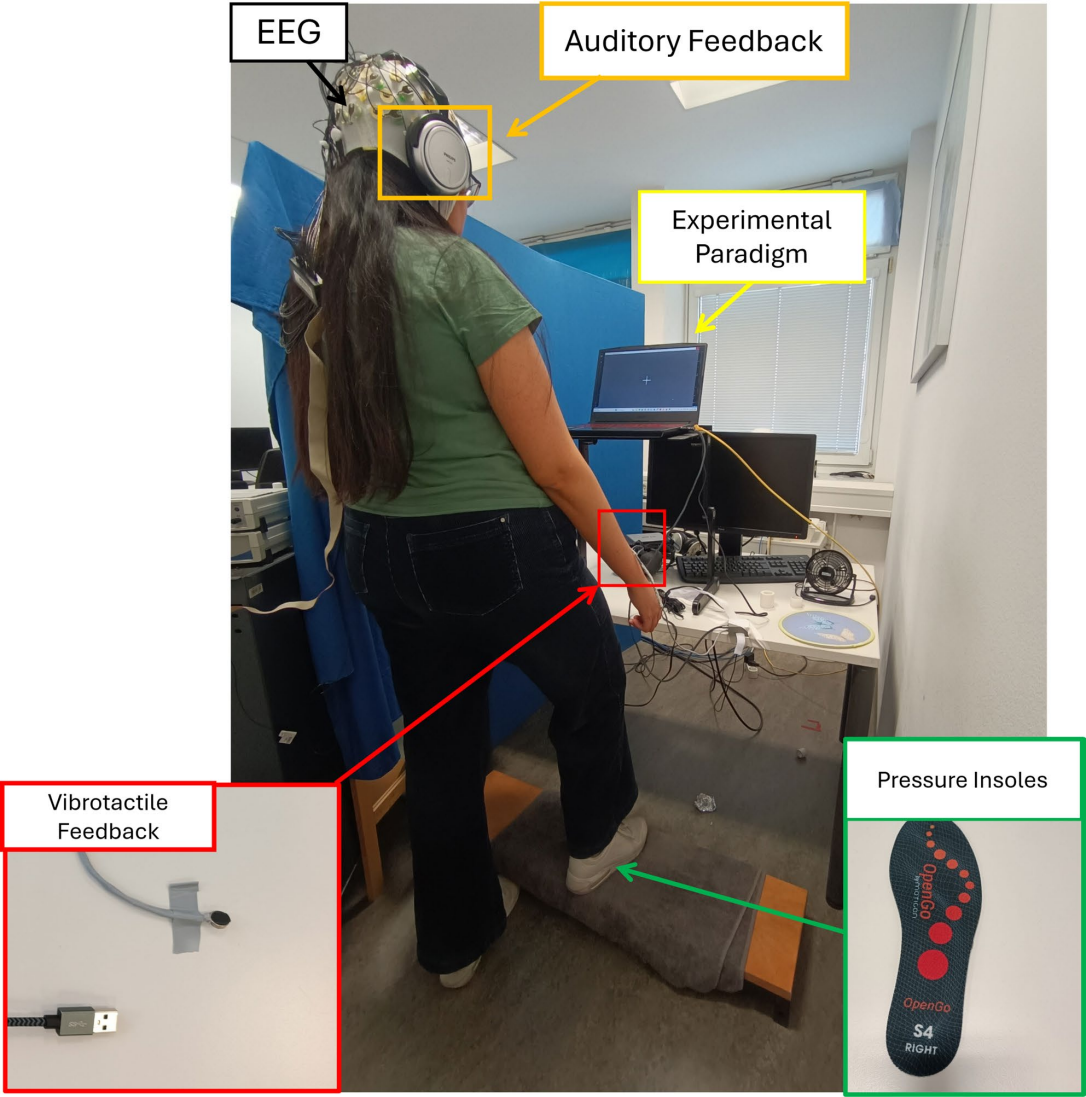
Experimental Setup. The participant is wearing an EEG cap with 32 electrodes and the pressure insoles inside their shoes while standing. The experimental paradigm is shown in a 16” screen. When the participant takes a step (lifting the dominant leg and putting it down on a wood step), they receive artificial sensory feedback. The auditory feedback is delivered via headphones to the dominant side. The vibrotactile feedback is delivered using a vibromotor placed on the dominant arm.

Figure 1 shows the setup for the experiment.

### EEG data acquisition, pre-processing, and analysis

The EEG data were recorded using the actiChamp amplifier and the 32-electrode system from Brain Products (Munich, Germany). The EEG signals were captured using Ag-AgCl active electrodes in an EEG cap, placed following the 10–20 International System to ensure the correct position of the electrodes over the brain areas of interest. The signals were digitized at 1000 Hz and no filters were applied during the signal recording. In addition, impedances were kept below 15 kOhms. The ground electrode was placed at FPz, and the signals were referenced to linked mastoid electrodes. The mastoid electrodes were placed directly over the mastoid bones using Ten20 electrode paste after cleaning the area with alcohol. Vertical eye movements were captured with the Fp1 and Fp2 channels.

The EEG data was pre-processed using custom Python scripts (version 3.11.1.) and the MNE Python library (version 1.7.0). The data was high-pass filtered with a 0.1 Hz filter, and low-pass filtered with a cutoff of 30 Hz. A 50 Hz notch filter (and harmonics 100, 150 and 200 Hz) was applied to filter out line noise. All filters were zero-phase, finite impulse response (FIR) filters designed using a Hamming window. Independent component analysis (ICA) was used to identify noise sources in the data, i.e. ocular, muscular, and electrical artifacts. For each participant and EEG recording, the artifact-related components were identified via visual inspection of the time courses and topographies. After removing these components, the data were reconstructed by back-projecting the remaining components.

The data from each condition were then segmented to create epochs time-locked to the onset of the auditory and vibrotactile stimulus. The epochs had a length of 1200 ms, covering a window from − 500 ms to 700 ms relative to the stimulus. A baseline correction from − 200 ms to 0 ms relative to the stimulus was applied, and epochs with a peak-to-peak amplitude exceeding 150µV were rejected, on average 3.07 ± 4.41 epochs. Lastly, we applied a temporal shift to the ERP data to correct the intrinsic delay in the stimulation apparatus. The temporal shift was 180 ms in the auditory condition and 29 ms in the vibrotactile modality.

ERP analysis of the N100 and P300 components was done on average for each condition. For the auditory stimulus, the average was calculated across the fronto-central electrodes Fz, FCz and Cz [20, 51, 53]. For the vibrotactile modality, the average amplitude was calculated across the left somatosensory cortex, electrodes C3, FC5, and CP5, as the vibrotactile stimulation was applied to the right arm. The N100 and P300 component amplitudes were extracted for further analysis for each participant and condition. For each ERP component, the amplitude was calculated as the average amplitude in a ± 25 ms window around the peak time. In the auditory modality, the N100 component was identified as the most negative value between 50 ms and 200 ms and the P300 component was identified as the most positive value between 250 ms and 350 ms. In the vibrotactile modality, the N100 component was identified between 100 ms and 250 ms, and the P300 component was identified between 250 ms and 350 ms.

### Explicit sense of agency ratings

Explicit Sense of Agency was measured through a four-question questionnaire using a 7-point Likert Scale from − 3 (“Strongly Disagree”) to 3 (“Strongly Agree”). The questionnaire was adjusted for each modality.

**Auditory modality**:

1. I felt that it was me who caused the sound.
2. Hearing a sound when taking a step felt natural.
3. I could clearly perceive the sound.
4. I felt in control over the sound.

**Vibrotactile modality**:

1. I felt that it was me who caused the vibration.
2. Receiving the vibration when taking a step felt natural.
3. I could clearly perceive the vibration.
4. I felt in control over the vibration.

For each modality, questions 1 and 4 were intended to give an assessment over the level of control participants experienced during each block. The rating of these two questions were used as the explicit SoA measures. Questions 2 and 3 were introduced as a manipulation check for the perception of the stimuli.

### Procedure and experimental protocol

The experimental session lasted approximately 120 min and was structured into sensory modality blocks, with short breaks between blocks. At the beginning of the session, the participant was informed about the experimental procedure and gave their informed written consent. Then the participant put on the pressure insoles and the experimenter set up the EEG system. Participants received verbal instructions before each experimental condition. In the P condition, participants were shown the paradigm window in which a grey background was shown. After an inter-trial interval of 1500 ms, a white fixation cross appeared on the screen. Then, after a random delay between 1000 and 1500 ms, the stimulus was delivered. In the OT condition, participants were shown the background image on the screen. After the inter-trial interval of 1500ms, the white fixation cross appeared on the screen and participants lifted their right leg onto a platform placed on the ground to simulate the action of taking a step. This stepping motion was detected using the pressure insoles and consisted of a liftoff and a step-down event. Following the step-down detection, the participant either heard the auditory tone or felt the vibrotactile stimulation (depending on the condition). The delay between the step-down event and the stimulus was fixed to be 200, 500 or 800 ms. After receiving the stimulation, participants were shown the rating scale on the screen and asked to estimate the time interval between stepping down and perceiving the stimulus. Lastly, in the IEB baseline condition, the participant was shown the paradigm window and after the inter-trial interval, the white fixation appeared on the screen. After an individualized delay based on the movement onset from the OT condition (mean ± standard deviation), the fixation cross briefly changed colour to red and the stimulus was delivered after the fixed 200, 500 or 800 ms.

**Fig. 2:**
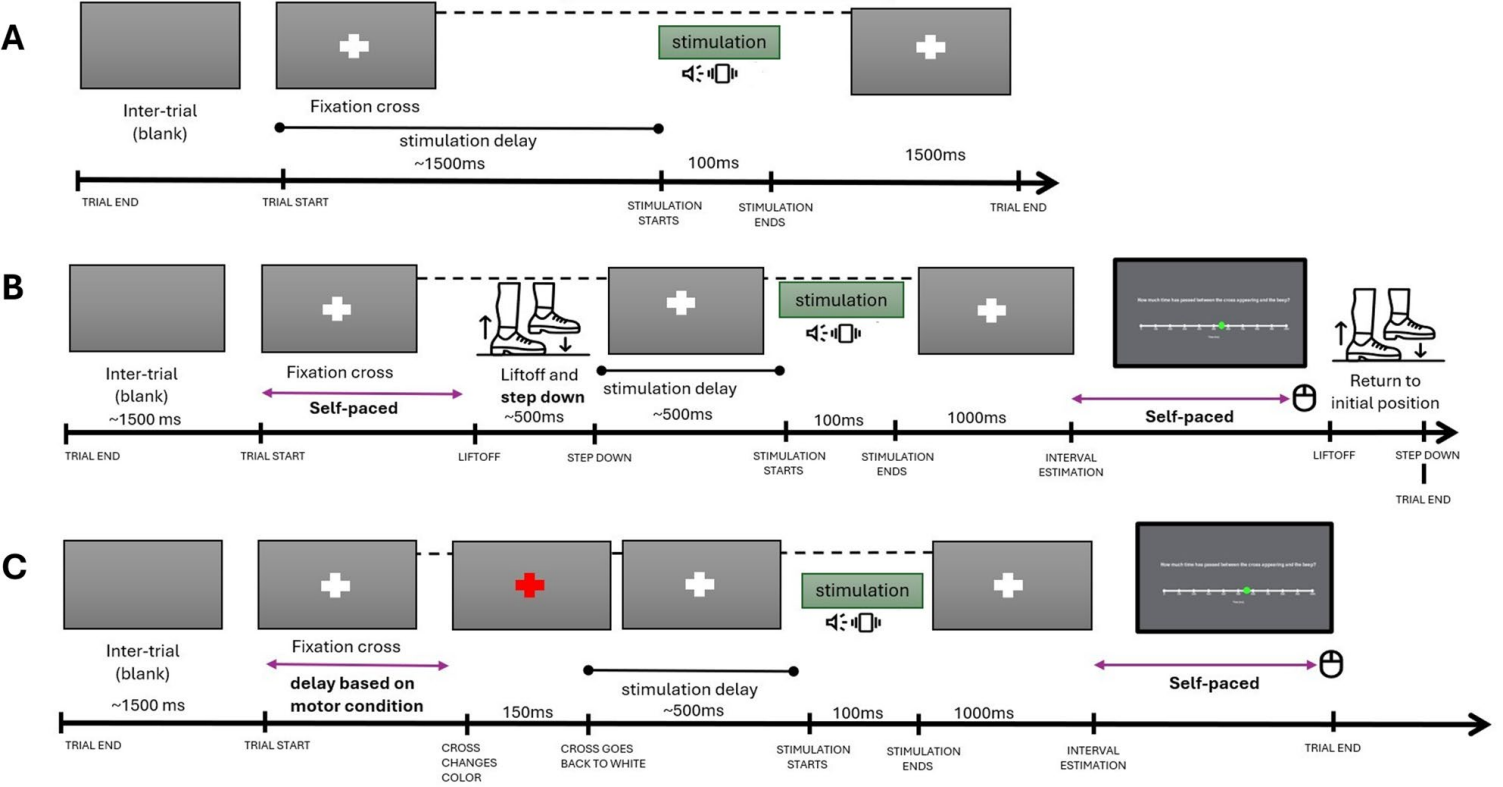
Experimental conditions. Panel** A** shows the trial structure of the passive condition. Panel** B** shows the trial structure of the operant task condition. Panel** C** shows the trial structure of the interval estimations baseline condition.

The participants did not have time restrictions to either perform the stepping movement or provide the interval estimation rating (Fig. 2).

### Statistical analysis

#### ERPs

The EEG data was analysed in Python. For each of the ERP components, the amplitudes were averaged across the selected channels for each modality. Significant effects between passive and operant task conditions were investigated using paired t-tests. The threshold for significance level was set to *p* < 0.05. Cohen’s D is calculated to report effect sizes for t-tests.

#### Temporal interval estimations

We performed a two-way repeated measures ANOVA test, with the experimental condition (IEB, operant task) and the stimulation delay (200, 500, and 800ms) as the within-subject factors. If necessary, we performed paired t-tests to examine post-hoc comparisons.

#### Explicit SoA reports

For easier interpretation, we transformed the SoA questionnaire answers from the Likert scale range (– 3, 3) to a 0 to 100 score scale where 0 represents no feeling of agency and 100 represents maximum feeling of agency. We performed a 1 sample t-test to assess if explicit SoA reports differed from a neutral score, i.e. participants are uncertain of whether the sensory feedback was self or externally generated.

## Results

### Vibrotactile modality

**Fig. 3:**
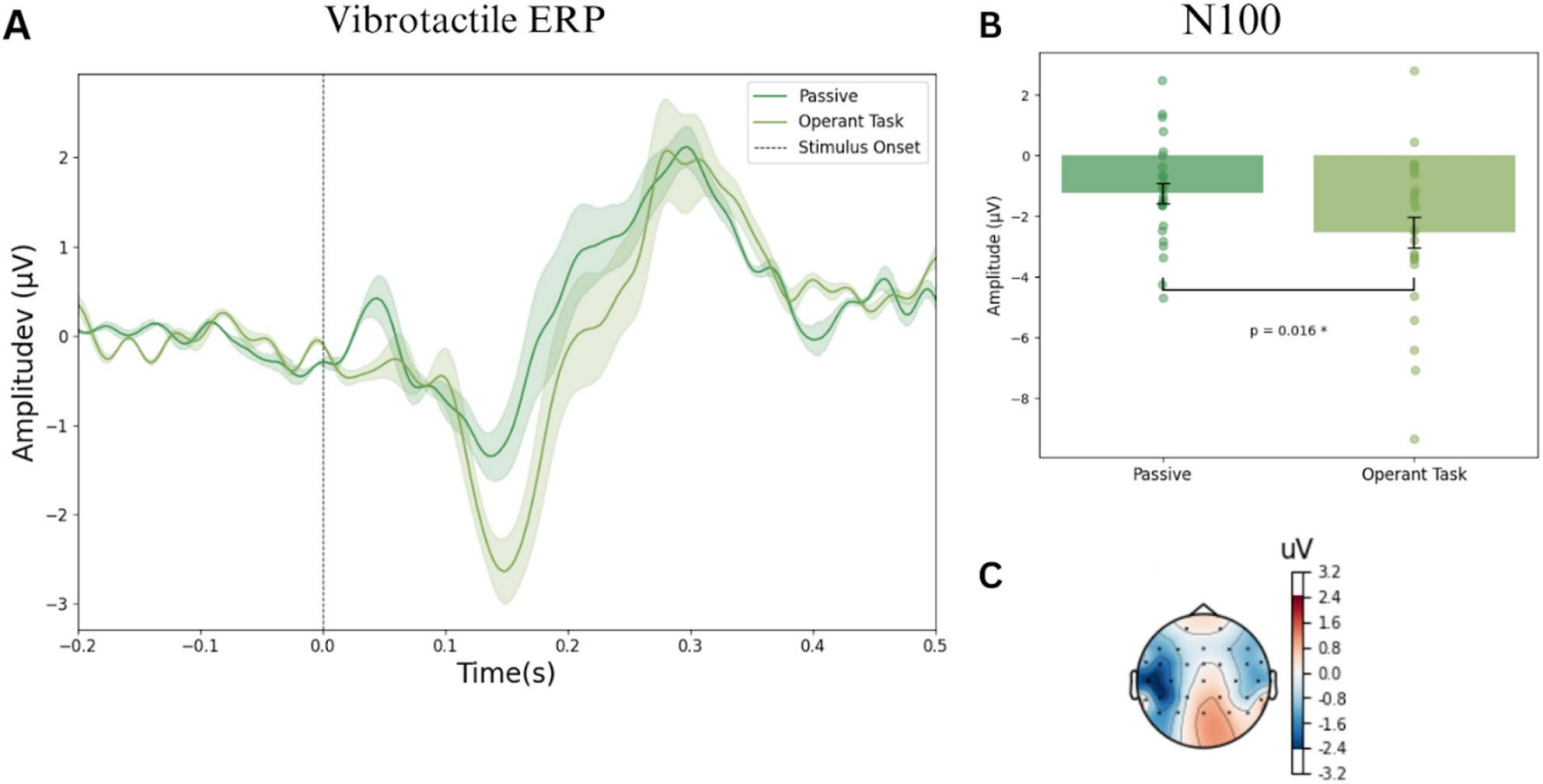
Vibrotactile ERPs. Panel A shows the grand average ERP across channels C3, FC5 and CP5 for each experimental condition, passive and operant task (*n* = 25). The grey highlighted area indicates the time window used for the analysis of each ERP component. Panel B shows the average N100 amplitude across channels C3, FC5 and CP5 for each experimental condition(*n* = 25). Error bars represent the mean ± standard error. Pairwise t-tests were two-tailed and statistical significance (*P* < 0.05) is represented with *. Panel C shows the scalp topography of the difference in amplitude between experimental conditions at timepoint 143.6ms, the N100 latency.

ERP analysis of the N100 component in the vibrotactile modality was performed using the average N100 amplitude across the channels C3, FC5 and CP5 in the time window of ± 25ms around the peak amplitude. Pairwise t-test showed a significant N100 amplitude difference between the passive (mean = – 1.23 µV, SE = 0.34µV) and operant task condition (mean = – 2.52 µV, SE = 0.51µV), with t(24) = 2.60, *P* = 0.016, and Cohen’s D = 0.58 (See Fig. 3).

**Fig. 4:**
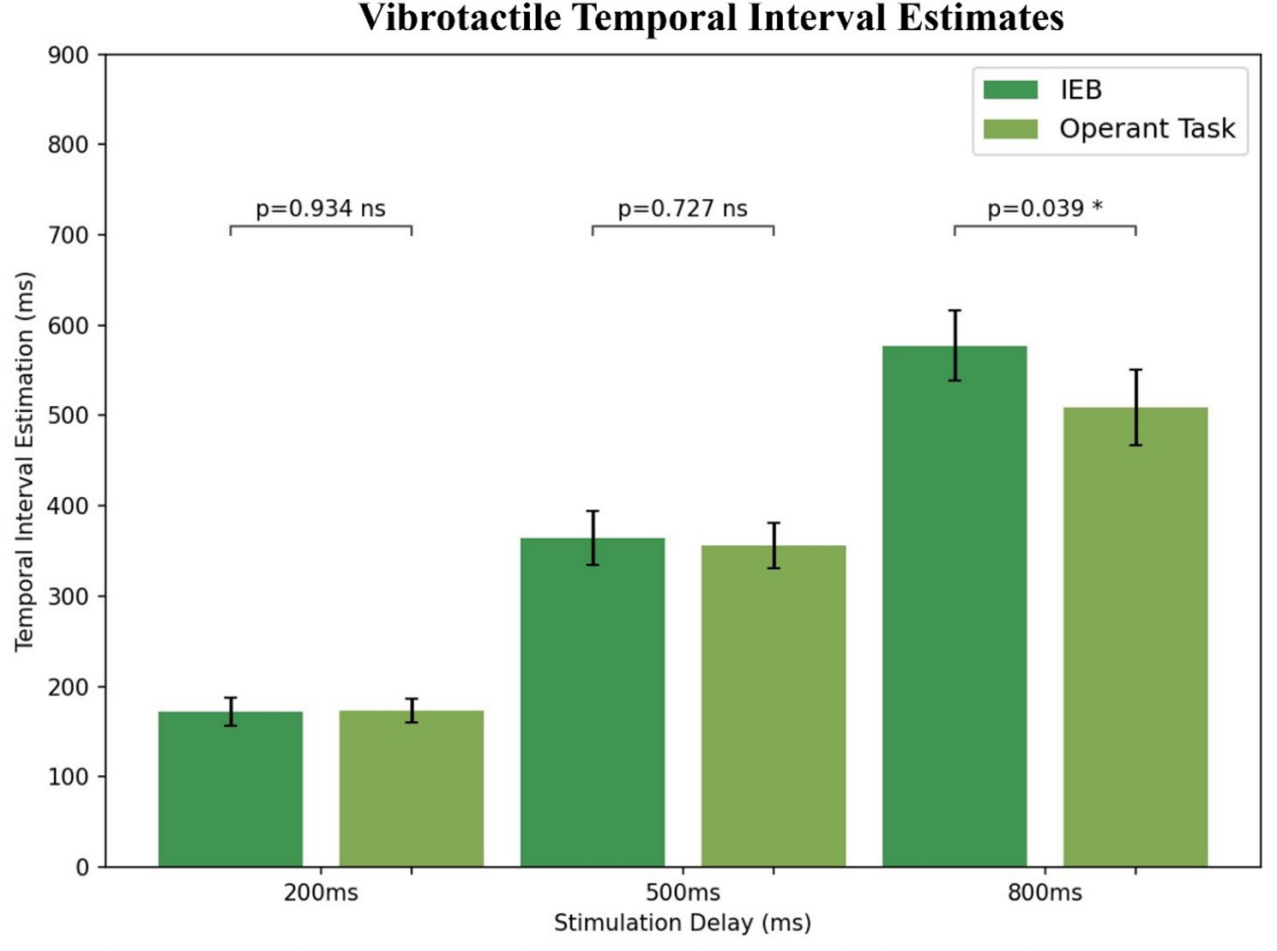
Vibrotactile Temporal interval Estimations for each experimental condition, separated by stimulation delay. Error bars represent the mean ± standard error. ns represents a non-significant difference in temporal interval estimates across experimental conditions.

Temporal interval estimates were analysed using a two-way ANOVA test that, similarly to the auditory condition, showed no effects between the IEB and the operant task conditions (*P* = 0.198, η^2^ = 0.007), and an effect on the stimulation delay (*P* < 0.001, η^2^ = 0.515) and an interaction effect between experimental condition and stimulation delay (*P* = 0.019, η^2^ = 0.011). Follow up t-tests showed no significant effect for the 200ms (t(24) = – 0.08, *P* = 0.934) or 500ms delay (t(24) = 0.35, *P* = 0.727), but it showed a significant effect in the 800ms delay (t(24) = 2.18, *P* = 0.039, Cohen’s D = 0.34) (See Fig. 4).

### Auditory modality

**Fig. 5:**
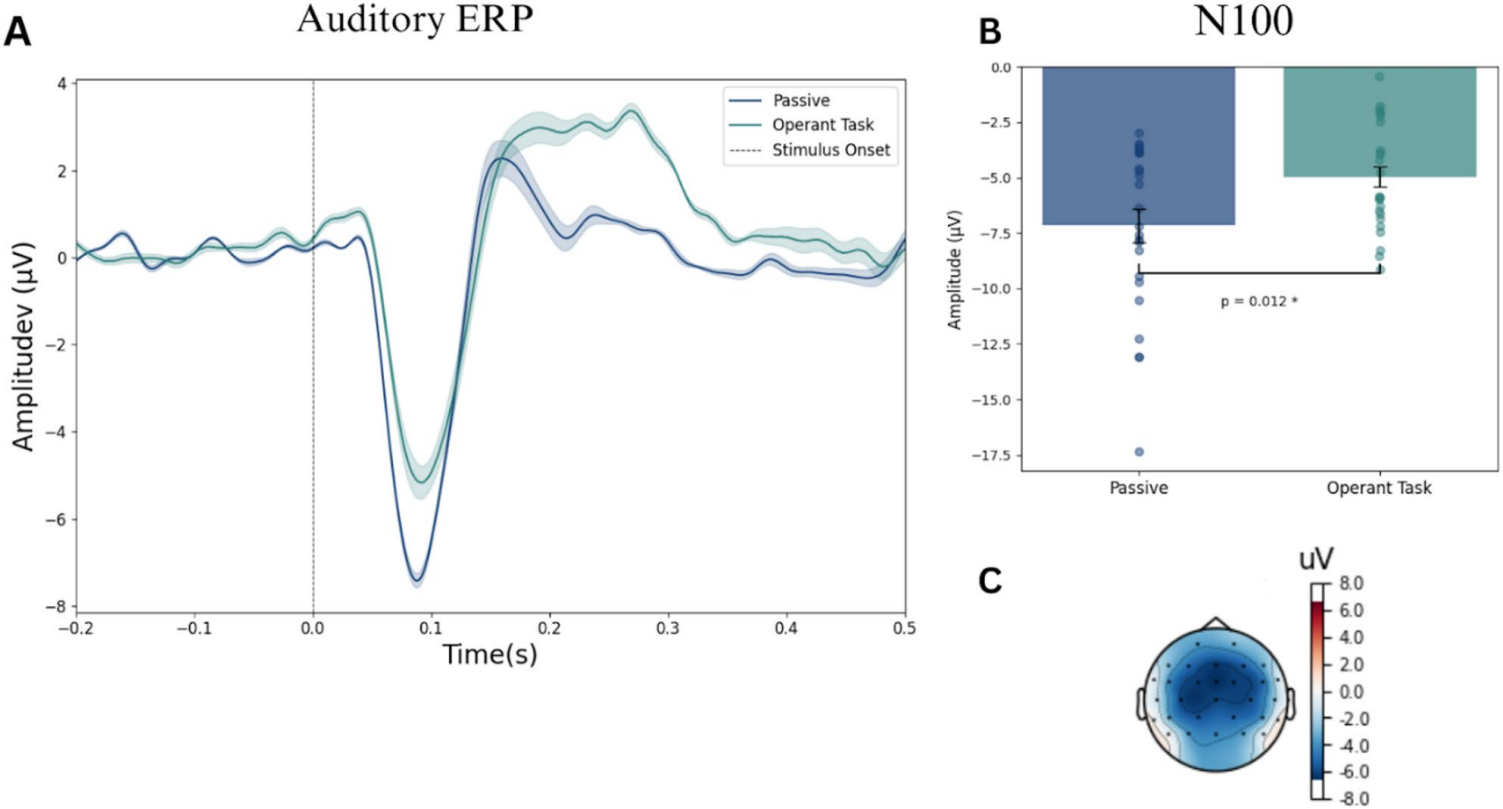
Auditory ERPs. Panel A shows the grand average ERP across channels Fz, FCz and Cz for each experimental condition, passive and operant task (*n* = 25). The grey highlighted area indicates the time window used for the analysis of each ERP component. Panel B shows the average N100 amplitude across channels Fz, FCz and Cz for each experimental condition (*n* = 25). Error bars represent the mean ± standard error. Pairwise t-tests were two-tailed and statistical significance (*P* < 0.05) is represented with *. Panel C shows the scalp topography of the difference in amplitude between experimental conditions at timepoint 89 ms.

ERP analysis of the N100 component was conducted using the average N100 amplitude across the channels Fz, FCz and Cz in a time window of ± 25ms around the peak amplitude. Pairwise t-test showed a significant difference in N100 amplitude between the passive condition (mean = – 7.16 µV, SE = 0.75µV) and the operant task condition (mean = – 4.95 µV, SE = 0.48µV), with t(24) = – 2.73, *P* = 0.012 and Cohen’s D = – 0.69 (See Fig. 5).

**Fig. 6:**
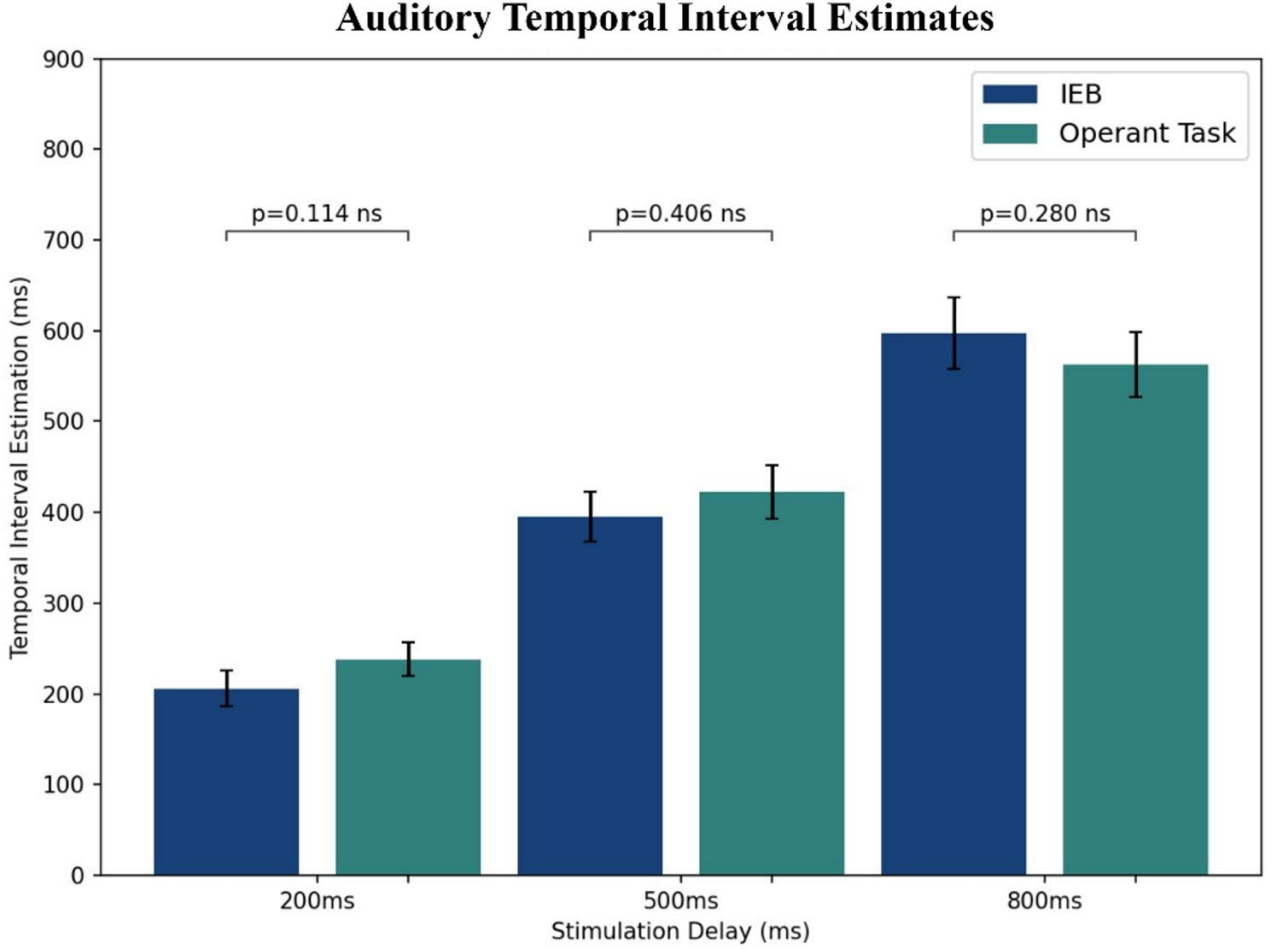
Auditory Temporal Interval Estimations for each experimental condition, separated by stimulation delay. Error bars represent the mean ± standard error. ns represents a non-significant difference in temporal interval estimates across experimental conditions.

In the temporal interval estimates, a two-way repeated measures ANOVA test showed no differences between the IEB and operant task conditions (*P* = 0.73, η^2^ = 0.0008). It also showed a main effect on the stimulation delay (*P* < 0.001, η^2^ = 0.496), indicating sensitivity to differentiate between stimulation delays. Lastly, it showed interaction effects between the experimental condition and the stimulation delay (*P* = 0.017, η^2^ = 0.01). Therefore, we conducted follow-up paired t-tests between experimental conditions for each stimulation delay. This resulted in no significant differences for the 200ms (t(24) = -1.64, *P* = 0.114), 500ms (t(24) = -0.85, *P* = 0.406), or 800ms delay (t(24) = 1.11, *P* = 0.280) (See Fig. 6).

### Explicit SoA results

We calculated the average rating score for questions 1, “I felt that it was me who caused the sound/vibration”, and 4, “I felt in control over the sound/vibration”, in the SoA questionnaire (*n* = 25) for auditory (mean = 57.33, STD = 26.71) and vibrotactile (mean = 53.33, STD = 26.68) modalities. A 1 sample t-test showed no significant difference from neutral rating (score = 50) for either sensory modality (t(24)_aud_ = 1.37, *P*_*aud*_ = 0.183; t(24)_vib_ =0.63, *P*_vib_ = 0.538).

### Exploratory analysis

**Fig. 7:**
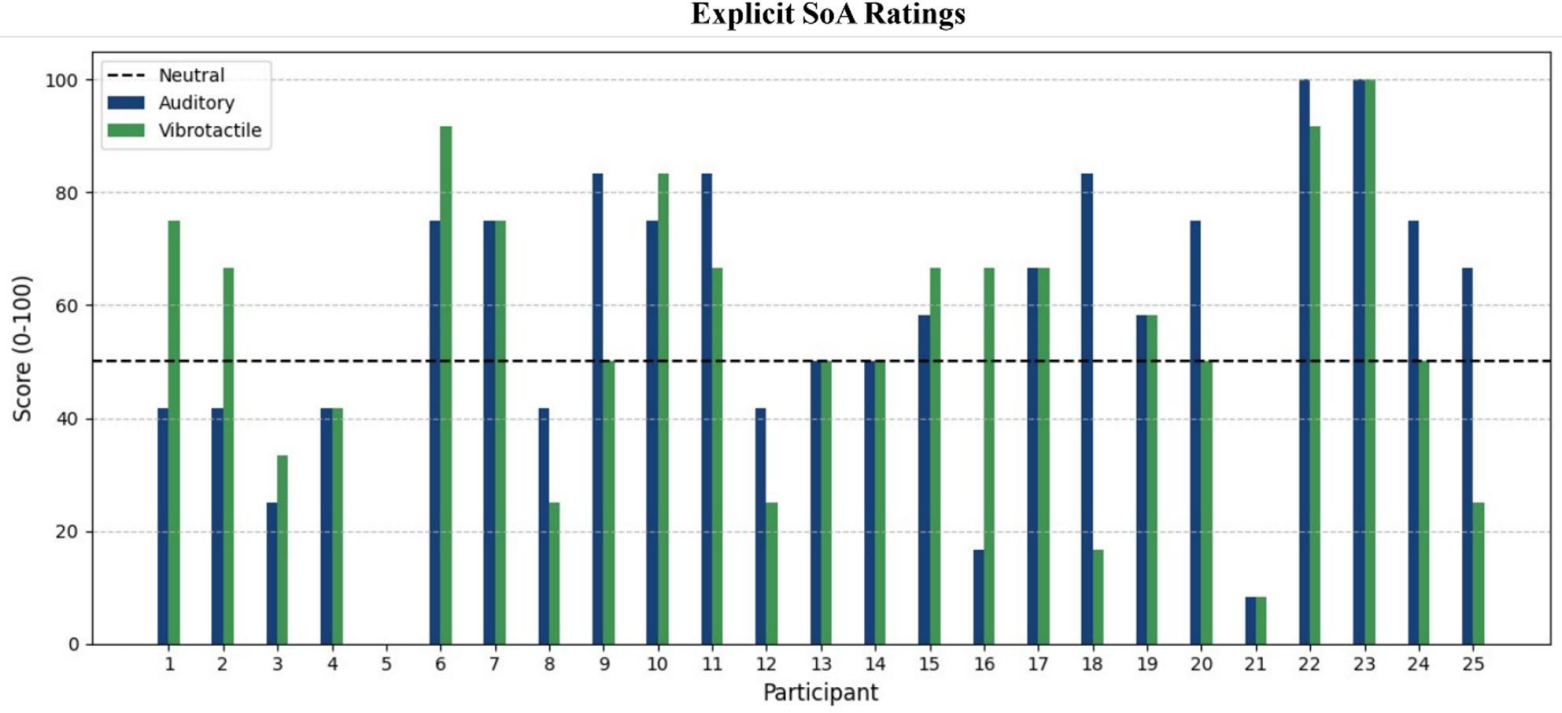
Explicit SoA Rating for each participant in the auditory (blue) and vibrotactile (vibrotactile) modalities. Bars represent the average score for questions 1 (“I felt that it was me who caused the sound/vibration”) and 4 (“I felt in control over the sound/vibration”) for each artificial sensory feedback modality in the SoA questionnaire.

To further explore the potential reasons why the explicit SoA judgment scores were very low and not significantly different from the “neutral” point on the scale, we examined the explicit scores at the individual participant level. After calculating the average SoA rating scores for each participant and modality and observing a large variance in scores (see Fig. 7), we were able to identify two SoA rating groups in each modality. One subgroup of participants reported experience of agency (rating score > 50), whereas another subgroup did not report experience of agency (rating score ≤ 50). In the auditory modality, 14 participants reported experience of agency and 11 reported uncertain or no feeling of agency, and a 1-sample t-test showed that each of the subgroups, a positive SoA rating (mean rating = 76.79, STD = 12.73) and neutral or negative SoA rating (mean rating = 32.58, STD = 17.26) differed significantly from the neutral point in the rating (t(13)_pos_ = 7.87, *P*_pos_ < 0.001; t(10)_neg_ = -3.34, *P*_neg_ = 0.007). In the vibrotactile modality 12 participants reported SoA, and a 1-sample t-test showed similar results, with significant differences between SoA rating and the neutral point for both the positive SoA rating group (mean rating = 75.69, STD = 13.04) and the neutral or negative SoA rating group (mean rating = 32.69, STD = 17.50) (t(11)_pos_ = 6.83, *P*_pos_ < 0.001; t(12)_neg_ = -3.57, *P*_neg_ = 0.004). These results suggest that individuals experience different levels of control under the same circumstances and should be further studied in future research.

### Auditory and vibrotactile P300 component

Additionally, based on the visual inspection of the grand average ERPs, we examined the late positive component in both auditory and vibrotactile sensory modality.

**Fig. 8:**
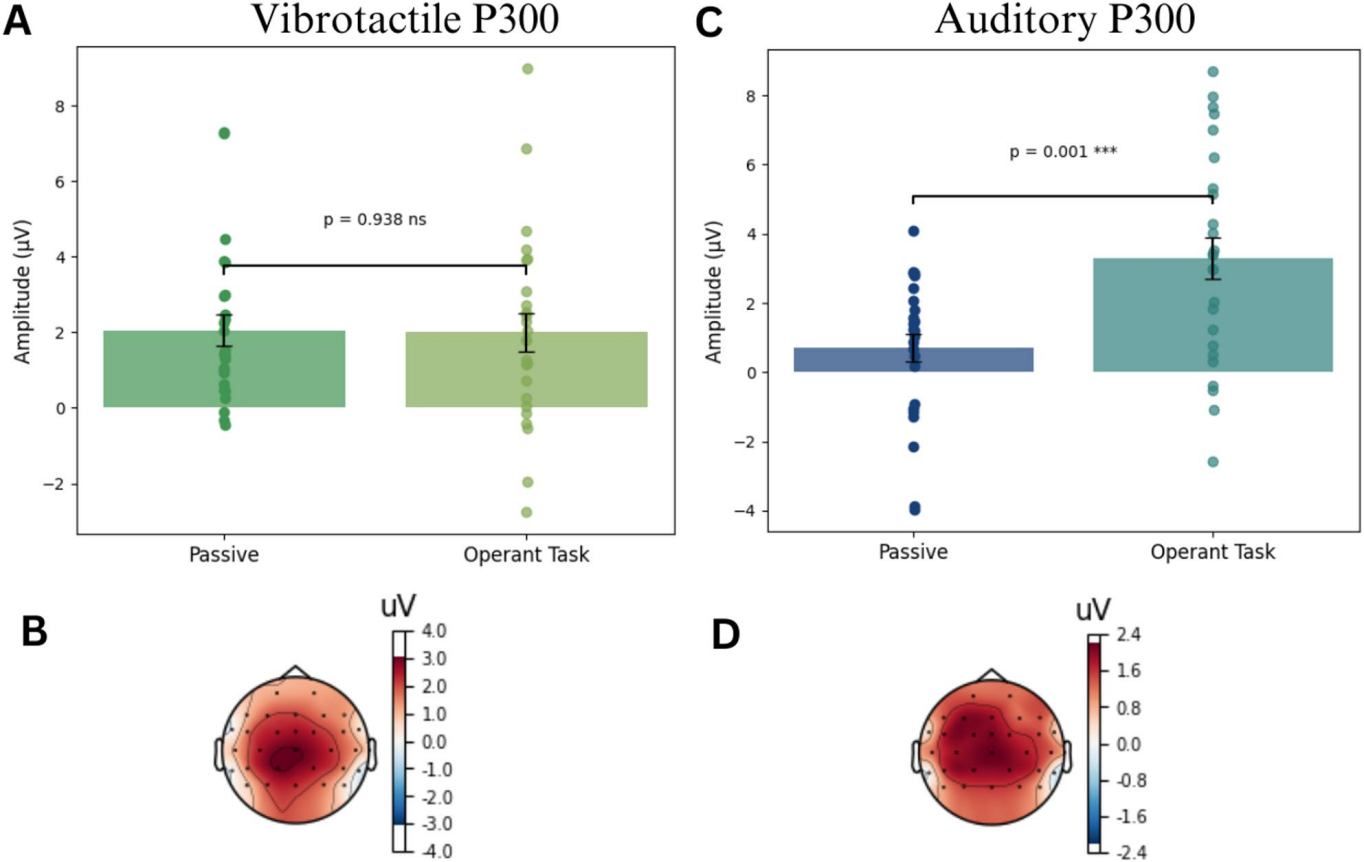
ERP late positive components. Panels A and B show the P300 component in the vibrotactile modality. Panel A shows the P300 amplitude across channels C3, FC5 and CP5 for each experimental condition(*n* = 25). Error bars represent the mean ± standard error. Pairwise t-tests were two-tailed and statistical significance (*P* < 0.05) is represented with *. Panel B shows the scalp topography of the difference in amplitude between experimental conditions at timepoint 298.6ms. Panels C and D are analogous to A and B, showing the P300 component in the auditory modality. Panel C shows the average P300 amplitude across channels Fz. FCz and Cz for each experimental condition (*n* = 25). Error bars represent the mean ± standard error. Pairwise t-tests were two-tailed and non-significant results are represented with ns. Panel D shows the scalp topography of the difference in amplitude between conditions at timepoint 267 ms.

In the vibrotactile modality, ERP analysis of the P300 component was performed using the average P300 amplitude across the channels C3, FC5 and CP5 in the time window of ± 25ms around the peak amplitude. Pairwise t-test showed no significant P300 amplitude difference between the passive (mean = 2.05µV, SE = 0.40µV) and operant task condition (mean = 2.00 µV, SE = 0.51µV). (See Fig. 8, A and B).

In the auditory modality, ERP analysis of the P300 component was performed using the average P300 amplitude across channels Fz, FCz and Cz in the time window of ± 25ms around the peak amplitude. Pairwise t-tests showed a significant difference between the passive (mean = 0.7 µV, SE = 0.41µV) and operant task (mean = 3.29µV, SE = 0.61µV), with t(24) = -3.76, *P* = 0.001, and Cohen’s D = -0.98 (see Fig. 8, C and D).

## Discussion

In this study, we aimed to validate a vibrotactile sensory feedback system with regards to its ability to elicit SoA. We designed a system and experimental paradigm that resembles naturalistic scenarios in which assistive systems with artificial sensory feedback are used.

Our results revealed a N100 modulation effect during the use of our vibrotactile sensory feedback system, i.e. in self-generated vibrotactile feedback. This indicates that participants’ brains reliably identified when they had control over the vibrotactile sensory feedback and adjusted sensory processing accordingly. To verify the use of sensory modulation as a suitable metric of SoA, we examined it in the control modality, auditory, where the expected outcome is well established. Here, we found a N100 attenuation effect, consistent with previous findings [20, 21, 47].

In contrast, explicit measures of SoA and temporal interval estimates did not exhibit an enhanced SoA in response to self-generated vs. externally generated stimuli. There was an exception in the case of the 800ms delay in the vibrotactile modality, where we could observe a significant difference. However, due to the small number of trials per stimulation delay and relatively small effect size, further research with a larger number of trials per participant would be required to determine the robustness of this finding.

In addition, we observed differences in their reports of explicit SoA. Some participants reported a stronger SoA, while under the same conditions, other participants reported no or weak SoA. These individual differences in SoA might be due to factors such as individual differences in sensitivity to control [54], or individual sensory modality dominance [55]. Individual difference in perceptual sensitivity to changes in control could explain why, under the same conditions, some participants experience SoA at a conscious level while others do not. Likewise, if some participants are more attuned to one sensory modality over another (e.g. auditory over vibrotactile), receiving sensory feedback in said modality could lead to a higher explicit SoA rating when compared to a participant with a different sensory modality preference. Further exploration of these individual differences would require a larger sample size and an experimental design specifically focused on exploration of individual differences.

### N100 modulation

Although we did not compare the auditory and vibrotactile sensory modalities directly (as the sensory stimulation is very different and thus, we believe not directly comparable), we observed differences in the neural responses in each modality. In the auditory modality, we observed a N100 attenuation in response to self-generated stimuli, whereas in the vibrotactile modality, we observed an N100 enhancement. Both sensory attenuation and enhancement of the neural responses in response to predictable (or self-generated) sensory stimuli have been reported in the literature. In the context of SoA, attenuation of N100 has been associated with an increased SoA [20–22, 56]. However, an enhancement of neural responses has also been observed before [23, 51, 57–59]. In these studies, researchers manipulated attention and predictability to investigate how they might affect sensory processing and found an interaction between the two in the N100 response [23, 51, 57] and in fMRI [58, 59]. Attention might determine the level of cortical responsiveness to the stimulus, with attended stimuli leading to enhanced neural responses and outweighing any suppression caused by predictability. Additionally, it is possible that the enhancement of N100 in the vibrotactile stimulus was due to the stimulus being more proximal than the auditory modality. Another possibility is that the vibrotactile stimulus was lower intensity and less salient than the auditory stimulus and required participants to be more attentive to it. Overall, however, it is important to point out that the neural responses to vibrotactile sensory modality are understudied and thus, we cannot formulate any conclusions regarding the directionality of the N100 modulation. Importantly, the difference in N100 amplitude between the passive and the operant condition (independent of the directionality) clearly demonstrates the brain’s sensitivity to self- vs. externally generated stimuli and thereby, confirms that the vibrotactile feedback was potent enough to elicit SoA in the participants of our study.

### Dissociation of implicit and explicit SoA measures

The observation of effects in sensory modulation but not in temporal interval estimates or explicit measures suggests that these metrics might be associated with separate processes underlying SoA, as previously proposed in the literature. Prior research has found that implicit and explicit measures of SoA were not correlated, thus suggesting that two separate processes might drive implicit SoA and explicit SoA measures [60]. This dissociation could be linked to predictive and postdictive mechanisms, which both give rise to the experience of agency. Predictive mechanisms, which rely on the internal forward model and the predicted sensory feedback [3], are fast and fundamental for pre-reflective experience of SoA [56, 61]. These predictive processes result in a rapid sense of control, based on sensorimotor integration and associated with neural implicit measures of SoA, i.e. modulation of sensory processing. This fast process is less influenced by cognitive factors, and it is less likely to be accessed consciously. On the other hand, postdictive processes are slower and higher-cognitive level than predictive processes. They occur after the action and its outcome have been completed and depend on the cognitive interpretations of events (sensory feedback in relation to the actions performed) retrieved already from memory. Postdictive processes likely contribute to explicit SoA judgements, which are reflective [62–64]. Wegner [64] suggested that agency is the result of *post-hoc* inference during and after the action has occurred, thus making these processes susceptible to influences from other cognitive mechanisms (including memory). Explicit SoA measures, like SoA questionnaires, require participants to consciously recall the experience of control they experienced under certain circumstances and are tightly linked to postdictive processes. Temporal interval estimations, although an implicit measure, also requires participants to recall the amount of time that passed between two events making it a rather postdictive process. Moreover, Siebertz & Jansen [32] found temporal interval estimations to be uncorrelated to measures obtained using the Libet clock paradigm, despite both paradigms having been extensively utilized to measure intentional binding before. This suggests that these paradigms may assess different aspects of SoA and highlights the need for further research to investigate their reliability. Together, this suggests that explicit SoA measures and temporal interval estimations are susceptible to influences from other cognitive mechanisms and manipulations and thus, not always reliable.

Predictive and postdictive mechanisms of SoA are not mutually exclusive but likely work together to elicit a strong SoA. Synofzik and colleagues [62] proposed that SoA arises from a complex interplay of predictive and postdictive processes. They suggested that the integration of internal and predictive cues and that the low-level, pre-reflective *feeling* of agency that results from the predictive process could lead to a more explicit *judgement* of agency at the cognitive level. This explains why we observed an effect in neural measures, N100 modulation, but not in the temporal interval estimations measure. Sensory modulation is sensitive to the low-level SoA, whereas temporal interval estimations and SoA questionnaires are linked to a higher order cognitive process. In other words, participants experience different degrees of SoA, which are reflected in the difference in N100 amplitude between conditions, but do not necessarily consciously experience agency, reflected in the lack of meaningful effects in temporal interval estimations and explicit judgements. In the design and use of assistive technologies, a strong low-level implicit SoA is required for optimal performance [65] as predictive mechanisms are crucial for efficient motor control through proper sensorimotor integration of actions produced with an artificial effector and artificial feedback delivered through the system. This can be achieved by ensuring minimal delays and congruent sensory feedback, and it can be measured using sensory modulation. This metric would be preferrable, as it has shown a higher sensitivity to implicit SoA in our study and thus, we recommend using this measure to address usability of the system and discriminate between agency and no agency conditions during use of sensory substitution systems or prosthesis. Additionally, explicit measures are oftentimes hard to acquire since it is not a continuous measure that can be obtained continuously throughout the task.

## Limitations

Our study was conducted using a relatively small number of trials to not exceed an experimental duration of two and a half hours. Additionally, we conducted the study with a population of healthy participants. Together, this might limit the generalizability of our findings, specifically in relation to clinical populations of individuals with sensory loss and/or prosthetic use. Future research should extend this study to clinical populations over extended time of use of the assistive system. Moreover, the intensity of the auditory and vibrotactile stimulus were not formally matched to ensure the perceived intensity was the same in both modalities. Future research, if aiming at comparing auditory with vibrotactile feedback, should match the salience of both signals through, for example, psychophysical tests measuring the Point of Subjective Equality.

## Future directions

This study was a first step towards a brain-in-the-loop approach for designing assistive technology that promote their user’s SoA. It also contributes to research efforts for identifying which measurement approaches are most appropriate in capturing SoA in naturalistic contexts. Future steps include further development of assistive technology including artificial sensory feedback and validation in clinical populations to understand whether such systems succeed in eliciting SoA. Additionally, individual differences in the explicit SoA measures were observed, implying different levels of sensitivity of SoA at a conscious level. Further differences between such two groups should be explored to better understand the mechanisms that lead to individuals consciously experiencing different levels of agency under similar circumstances.

## Conclusion

In summary, our findings indicate that employing vibrotactile sensory feedback in assistive technology successfully elicits SoA in its users. We observed a clear N100 component evoked from vibrotactile stimulus, which served as a sensory outcome of a lower-limb motor action. Importantly, this N100 component was significantly modulated by self-generated actions relative to the passive baseline condition, even though the directionality of this effect was opposite to the auditory modality, which might be explained, for example, by the proximity of the feedback or its attentional salience.

In addition, our study supports the argument that modulation of sensory processing is a sensitive measure of SoA. Moreover, the mixed effects in temporal interval estimates and explicit SoA highlight the likely dissociation between implicit and explicit SoA measures, presumably capturing different processes underlying the formation of SoA. Additionally, neural implicit measures have been shown to be a more adequate method to assess SoA in sensory substitution because it targets lower-level predictive processes and allows researchers to test their systems in real-world scenarios without having to use questionnaires after every experimental trial or block. Crucially, the use of sensory processing modulation instead of temporal interval estimations removes the need to manipulate the timing of sensory feedback, which is detrimental to the testing of sensory substitution systems, in which the target is to assess the system as it is, without added temporal delays.

In conclusion, our study suggests that vibrotactile feedback is a suitable sensory feedback modality to be used in assistive technologies. Vibrotactile sensory feedback is non-intrusive for the users and easy to incorporate into wearable systems. Furthermore, our study indicates N100 modulation could be a promising approach for evaluating the neuro-cognitive effects of providing artificial sensory feedback due to its sensitivity to agency and no agency conditions. This has important implications for the design and validation of future sensory substitution systems, prostheses, and other assistive technologies.

## Data Availability

Data will be available upon reasonable request.

## References

[1] Haggard P. Sense of agency in the human brain. Nat Rev Neurosci. 2017;18(4):196–207. 10.1038/nrn.2017.14. 28251993 10.1038/nrn.2017.14

[2] Zbinden J, Lendaro E, Ortiz-Catalan M. Prosthetic embodiment: systematic review on definitions, measures, and experimental paradigms. J Neuroeng Rehabil. 2022;19(1):37. 10.1186/s12984-022-01006-6. 35346251 10.1186/s12984-022-01006-6 PMC8962549

[3] Frith CD, Blakemore S-J, Wolpert DM. Explaining the symptoms of schizophrenia: abnormalities in the awareness of action. Brain Res Rev. 2000;31(2):357–63. 10.1016/S0165-0173(99)00052-1. 10719163 10.1016/s0165-0173(99)00052-1

[4] Blakemore S-J, Wolpert DM, Frith CD. Abnormalities in the awareness of action. Trends Cogn Sci. 2002;6(6):237–42. 10.1016/S1364-6613(02)01907-1. 12039604 10.1016/s1364-6613(02)01907-1

[5] Hughes G, Desantis A, Waszak F. Mechanisms of intentional binding and sensory attenuation: the role of Temporal prediction, Temporal control, identity prediction, and motor prediction. Psychol Bull. 2013b;139(1):133–51. 10.1037/a0028566. 22612280 10.1037/a0028566

[6] Blakemore S-J, Wolpert DM, Frith CD. Central cancellation of self-produced tickle sensation. Nat Neurosci. 1998a;1(7):635–40. 10.1038/2870. 10196573 10.1038/2870

[7] Friston K, Kiebel S. Predictive coding under the free-energy principle. Philos Trans R Soc Lond B Biol Sci. 2009;364(1521):1211–21. 10.1098/rstb.2008.0300. 19528002 10.1098/rstb.2008.0300 PMC2666703

[8] Shergill SS, Samson G, Bays PM, Frith CD, Wolpert DM. Evidence for sensory prediction deficits in schizophrenia. Am J Psychiatry. 2005;162(12):2384–6. 10.1176/appi.ajp.162.12.2384. 16330607 10.1176/appi.ajp.162.12.2384

[9] Wolpe N, Ingram JN, Tsvetanov KA, Geerligs L, Kievit RA, Henson RN, Wolpert DM, Rowe JB. Ageing increases reliance on sensorimotor prediction through structural and functional differences in frontostriatal circuits. Nat Commun. 2016;7(1):13034. 10.1038/ncomms13034. 27694879 10.1038/ncomms13034 PMC5063954

[10] Myers JC, Mock JR, Golob EJ. Sensorimotor integration can enhance auditory perception. Sci Rep. 2020;10(1):1496. 10.1038/s41598-020-58447-z. 32001755 10.1038/s41598-020-58447-z PMC6992622

[11] Reznik D, Henkin Y, Levy O, Mukamel R. Perceived loudness of Self-Generated sounds is differentially modified by expected sound intensity. PLoS ONE. 2015;10(5):e0127651. 10.1371/journal.pone.0127651. 25992603 10.1371/journal.pone.0127651 PMC4436370

[12] Blakemore S-J, Wolpert DM, Frith CD. Central cancellation of self-produced tickle sensation. Nat Neurosci. 1998b;1(7):635–40. 10.1038/2870. 10196573 10.1038/2870

[13] Kilteni K, Ehrsson HH. Functional connectivity between the cerebellum and somatosensory areas implements the Attenuation of Self-Generated touch. J Neurosci. 2020;40(4):894–906. 10.1523/JNEUROSCI.1732-19.2019. 31811029 10.1523/JNEUROSCI.1732-19.2019 PMC6975290

[14] Luck SJ. An introduction to the Event-Related potential Technique, second edition. MIT Press; 2014.

[15] D’Alonzo M, Cipriani C. Vibrotactile sensory substitution elicits feeling of ownership of an alien hand. PLoS ONE. 2012;7(11):e50756. 10.1371/journal.pone.0050756. 23226375 10.1371/journal.pone.0050756 PMC3511354

[16] Hughes G, Waszak F. ERP correlates of action effect prediction and visual sensory Attenuation in voluntary action. NeuroImage. 2011;56(3):1632–40. 10.1016/j.neuroimage.2011.02.057. 21352924 10.1016/j.neuroimage.2011.02.057

[17] Schafer EWP, Marcus MM. Self-Stimulation alters human sensory brain responses. Science. 1973;181(4095):175–7. 10.1126/science.181.4095.175. 4711735 10.1126/science.181.4095.175

[18] Niu N, Wu Y, Li H, Li M, Yang D, Fan W, Zhong Y. Influence of voluntary action and outcome Valence on the sense of agency. Front Hum Neurosci. 2023;17:1206858. 10.3389/fnhum.2023.1206858. 37746056 10.3389/fnhum.2023.1206858 PMC10512953

[19] Vastano R, Ambrosini E, Ulloa JL, Brass M. Action selection conflict and intentional binding: an ERP study. Cortex. 2020;126:182–99. 10.1016/j.cortex.2020.01.013. 32088407 10.1016/j.cortex.2020.01.013

[20] Harrison AW, Mannion DJ, Jack BN, Griffiths O, Hughes G, Whitford TJ. Sensory Attenuation is modulated by the contrasting effects of predictability and control. NeuroImage. 2021;237:118103. 10.1016/j.neuroimage.2021.118103. 33957233 10.1016/j.neuroimage.2021.118103

[21] Poonian SK, McFadyen J, Ogden J, Cunnington R. Implicit agency in observed actions: evidence for N1 suppression of tones caused by Self-made and observed actions. J Cogn Neurosci. 2015;27(4):752–64. 10.1162/jocn_a_00745. 25321488 10.1162/jocn_a_00745

[22] Timm J, SanMiguel I, Keil J, Schröger E, Schönwiesner M. Motor intention determines sensory Attenuation of brain responses to self-initiated sounds. J Cogn Neurosci. 2014;26(7):1481–9. 24392902 10.1162/jocn_a_00552

[23] Kaiser J, Schütz-Bosbach S. Sensory Attenuation of self-produced signals does not rely on self-specific motor predictions. Eur J Neurosci. 2018;47(11):1303–10. 10.1111/ejn.13931. 29738617 10.1111/ejn.13931

[24] Kiepe F, Hesselmann G. Sensory Attenuation of self-initiated tactile feedback is modulated by stimulus strength and Temporal delay in a virtual reality environment. Q J Experimental Psychol. 2025;17470218251330237. 10.1177/17470218251330237. 10.1177/17470218251330237 40087903

[25] Kaiser J, Buciuman M, Gigl S, Gentsch A, Schütz-Bosbach S. The interplay between affective processing and sense of agency during action regulation: a review. Front Psychol. 2021;12:716220. 10.3389/fpsyg.2021.716220. 34603140 10.3389/fpsyg.2021.716220 PMC8481378

[26] Moore JW. (2016). What Is the Sense of Agency and Why Does it Matter? *Frontiers in Psychology*, *7*. 10.3389/fpsyg.2016.01272 10.3389/fpsyg.2016.01272 PMC5002400 27621713

[27] Wen W, Imamizu H. The sense of agency in perception, behaviour and human–machine interactions. Nat Reviews Psychol. 2022;1(4):211–22. 10.1038/s44159-022-00030-6.

[28] Cesari V, D’Aversa S, Piarulli A, Melfi F, Gemignani A, Menicucci D. Sense of agency and skills learning in Virtual-Mediated environment: A systematic review. Brain Sci. 2024;14(4). 10.3390/brainsci14040350. 10.3390/brainsci14040350 PMC11048251 38672002

[29] Dewey JA, Knoblich G. Do implicit and explicit measures of the sense of agency measure the same thing? PLoS ONE. 2014;9(10):e110118. 10.1371/journal.pone.0110118. 25330184 10.1371/journal.pone.0110118 PMC4199671

[30] Qu J, Ma K, Hommel B. Cognitive load dissociates explicit and implicit measures of body ownership and agency. Psychon Bull Rev. 2021;28(5):1567–78. 10.3758/s13423-021-01931-y. 34033062 10.3758/s13423-021-01931-y

[31] Haggard P, Clark S, Kalogeras J. Voluntary action and conscious awareness. Nat Neurosci. 2002;5(4):382–5. 10.1038/nn827. 11896397 10.1038/nn827

[32] Siebertz M, Jansen P. Diverging implicit measurement of sense of agency using interval Estimation and libet clock. Conscious Cogn. 2022;99:103287. 10.1016/j.concog.2022.103287. 35131542 10.1016/j.concog.2022.103287

[33] Bekrater-Bodmann R. Factors associated with prosthesis embodiment and its importance for prosthetic satisfaction in lower limb amputees. Front Neurorobotics. 2021;14. 10.3389/fnbot.2020.604376. 10.3389/fnbot.2020.604376 PMC7843383 33519413

[34] Cornelio P, Haggard P, Hornbaek K, Georgiou O, Bergström J, Subramanian S, Obrist M. The sense of agency in emerging technologies for human–computer integration: A review. Front NeuroSci. 2022;16. 10.3389/fnins.2022.949138. 10.3389/fnins.2022.949138 PMC9511170 36172040

[35] Schofield JS, Shell CE, Thumser ZC, Beckler DT, Nataraj R, Marasco PD. Characterization of the sense of agency over the actions of Neural-machine Interface-operated prostheses. J Visualized Experiments: JoVE. 2019;143. 10.3791/58702. 10.3791/58702 30663709

[36] Bach-y-Rita P. Brain plasticity as a basis of sensory substitution. J Neurologic Rehabilitation. 1987;1(2):67–71. 10.1177/136140968700100202.

[37] Wright TD, Ward J. (2018). Sensory Substitution Devices as Advanced Sensory Tools. In F. Macpherson & F. Macpherson, editors, *Sensory Substitution and Augmentation* (p. 0). British Academy. 10.5871/bacad/9780197266441.003.0012

[38] Clemente F, Valle G, Controzzi M, Strauss I, Iberite F, Stieglitz T, Granata G, Rossini PM, Petrini F, Micera S, Cipriani C. Intraneural sensory feedback restores grip force control and motor coordination while using a prosthetic hand. J Neural Eng. 2019;16(2):026034. 10.1088/1741-2552/ab059b. 30736030 10.1088/1741-2552/ab059b

[39] Marasco PD, Hebert JS, Sensinger JW, Shell CE, Schofield JS, Thumser ZC, Nataraj R, Beckler DT, Dawson MR, Blustein DH, Gill S, Mensh BD, Granja-Vazquez R, Newcomb MD, Carey JP, Orzell BM. Illusory movement perception improves motor control for prosthetic hands. Sci Transl Med. 2018;10(432):eaao6990. 10.1126/scitranslmed.aao6990. 29540617 10.1126/scitranslmed.aao6990 PMC5906050

[40] Biddiss E, Beaton D, Chau T. Consumer design priorities for upper limb prosthetics. Disabil Rehabilitation: Assist Technol. 2007;2(6):346–57. 10.1080/17483100701714733. 10.1080/17483100701714733 19263565

[41] Pilger L, Berberich N, Paredes-Acuña N, Dendorfer A, Guadarrama-Olvera JR, Bergner F, Utpadel-Fischler D, Cheng G. Human-centered design of a vibrotactile sensory substitution belt for feet somatosensation in a patient with multiple sclerosis. 2023 11th International IEEE/EMBS Conference on Neural Engineering (NER), 2023, 1–4. 10.1109/NER52421.2023.10123871

[42] Ntakolia C, Dimas G, Iakovidis DK. User-centered system design for assisted navigation of visually impaired individuals in outdoor cultural environments. Univ Access Inf Soc. 2022;21(1):249–74. 10.1007/s10209-020-00764-1.

[43] Choi JS, Kim JG, Cho JH, Tack GR. A basic study on the effects of vibrator-attached leg-press on the knee and ankle joint torques. J Mech Med Biology. 2020;20(09):2040016. 10.1142/S0219519420400163.

[44] Cuppone AV, Squeri V, Semprini M, Masia L, Konczak J. Robot-Assisted proprioceptive training with added Vibro-Tactile feedback enhances somatosensory and motor performance. PLoS ONE. 2016;11(10):e0164511. 10.1371/journal.pone.0164511. 27727321 10.1371/journal.pone.0164511 PMC5058482

[45] Seim CE, Wolf SL, Starner TE. Wearable vibrotactile stimulation for upper extremity rehabilitation in chronic stroke: clinical feasibility trial using the VTS glove. J Neuroeng Rehabil. 2021;18(1):14. 10.1186/s12984-021-00813-7. 33485371 10.1186/s12984-021-00813-7 PMC7824932

[46] Bäß P, Jacobsen T, Schröger E. Suppression of the auditory N1 event-related potential component with unpredictable self-initiated tones: evidence for internal forward models with dynamic stimulation. Int J Psychophysiol. 2008;70(2):137–43. 10.1016/j.ijpsycho.2008.06.005. 18627782 10.1016/j.ijpsycho.2008.06.005

[47] Hughes G, Desantis A, Waszak F. Attenuation of auditory N1 results from identity-specific action-effect prediction. Eur J Neurosci. 2013a;37(7):1152–8. 10.1111/ejn.12120. 23331545 10.1111/ejn.12120

[48] Ea C, Jf C, A, C., P H. Coercion changes the sense of agency in the human brain. Curr Biology: CB. 2016;26(5). 10.1016/j.cub.2015.12.067. 10.1016/j.cub.2015.12.067 PMC4791480 26898470

[49] Ea C, Pa SLB, et al. The effect of military training on the sense of agency and outcome processing. Nat Communi 11(1). 10.1038/s41467-020-18152-x 10.1038/s41467-020-18152-x PMC7459288 32868764

[50] Jenkins M, Esemezie O, Lee V, Mensingh M, Nagales K, Obhi Ss. An investigation of ‘We’ agency in co-operative joint actions. Psychol Res. 2021;85(8). 10.1007/s00426-020-01462-6. 10.1007/s00426-020-01462-6 33398449

[51] Navare UP, Ciardo F, Kompatsiari K, De Tommaso D, Wykowska A. When performing actions with robots, attribution of intentionality affects the sense of joint agency. Sci Rob. 2024;9(91). 10.1126/scirobotics.adj3665. 10.1126/scirobotics.adj3665 38924424

[52] Engbert K, Wohlschläger A, Haggard P. Who is causing what? The sense of agency is relational and efferent-triggered. Cognition. 2008;107(2):693–704. 10.1016/j.cognition.2007.07.021. 17825813 10.1016/j.cognition.2007.07.021

[53] Ghio M, Scharmach K, Bellebaum C. ERP correlates of processing the auditory consequences of own versus observed actions. Psychophysiology. 2018;55(6):e13048. 10.1111/psyp.13048. 29266338 10.1111/psyp.13048

[54] Wen W, Ishii H, Ohata R, Yamashita A, Asama H, Imamizu H. Perception and control: individual difference in the sense of agency is associated with learnability in sensorimotor adaptation. Sci Rep. 2021;11(1):20542. 10.1038/s41598-021-99969-4. 34654878 10.1038/s41598-021-99969-4 PMC8519916

[55] Matsumoto M, Sakurada T, Yamamoto S. Distinct bilateral prefrontal activity patterns associated with the qualitative aspect of working memory characterized by individual sensory modality dominance. PLoS ONE. 2020;15(8):e0238235. 10.1371/journal.pone.0238235. 32845925 10.1371/journal.pone.0238235 PMC7449398

[56] Bays PM, Flanagan JR, Wolpert DM. Attenuation of Self-Generated tactile sensations is Predictive, not postdictive. PLoS Biol. 2006;4(2):e28. 10.1371/journal.pbio.0040028. 16402860 10.1371/journal.pbio.0040028 PMC1334241

[57] Hsu Y-F, Hämäläinen JA, Waszak F. Both attention and prediction are necessary for adaptive neuronal tuning in sensory processing. Front Hum Neurosci. 2014;8:152. 10.3389/fnhum.2014.00152. 24723871 10.3389/fnhum.2014.00152 PMC3972470

[58] Kok P, Rahnev D, Jehee JFM, Lau HC, de Lange FP. (2012). Attention reverses the effect of prediction in silencing sensory signals. *Cerebral Cortex (New York, N.Y.: 1991)*, *22*(9), 2197–2206. 10.1093/cercor/bhr310 10.1093/cercor/bhr310 22047964

[59] Reznik D, Ossmy O, Mukamel R. Enhanced auditory evoked activity to Self-Generated sounds is mediated by primary and supplementary motor cortices. J Neurosci. 2015;35(5):2173–80. 10.1523/JNEUROSCI.3723-14.2015. 25653372 10.1523/JNEUROSCI.3723-14.2015 PMC6705360

[60] Saito N, Takahata K, Murai T, Takahashi H. Discrepancy between explicit judgement of agency and implicit feeling of agency: implications for sense of agency and its disorders. Conscious Cogn. 2015;37:1–7. 10.1016/j.concog.2015.07.011. 26253893 10.1016/j.concog.2015.07.011

[61] Kahl S, Kopp S. A predictive processing model of perception and action for Self-Other distinction. Front Psychol. 2018;9. 10.3389/fpsyg.2018.02421. 10.3389/fpsyg.2018.02421 PMC6287016 30559703

[62] Synofzik M, Vosgerau G, Voss M. The experience of agency: an interplay between prediction and postdiction. Front Psychol. 2013;4:127. 10.3389/fpsyg.2013.00127. 23508565 10.3389/fpsyg.2013.00127 PMC3597983

[63] Takahata K, Takahashi H, Maeda T, Umeda S, Suhara T, Mimura M, Kato M. It’s not my fault: postdictive modulation of intentional binding by monetary gains and losses. PLoS ONE. 2012;7(12):e53421. 10.1371/journal.pone.0053421. 23285293 10.1371/journal.pone.0053421 PMC3532346

[64] Wegner DM. The mind’s best trick: how we experience conscious will. Trends Cogn Sci. 2003;7(2):65–9. 10.1016/S1364-6613(03)00002-0. 12584024 10.1016/s1364-6613(03)00002-0

[65] Nataraj R, Sanford S, Shah A, Liu M. Agency and performance of Reach-to-Grasp with modified control of a virtual hand: implications for rehabilitation. Front Hum Neurosci. 2020;14. 10.3389/fnhum.2020.00126. 10.3389/fnhum.2020.00126 PMC7191072 32390812

